# Uptake of Inorganic and Organic Nitrogen Sources by *Dinophysis acuminata* and *D. acuta*

**DOI:** 10.3390/microorganisms8020187

**Published:** 2020-01-29

**Authors:** María García-Portela, Beatriz Reguera, Jesús Gago, Mickael Le Gac, Francisco Rodríguez

**Affiliations:** 1 Spanish Institute of Oceanography (IEO), Oceanographic Center of Vigo, Subida a Radio Faro 50, Cabo Estay, Canido, 36390 Vigo, Spain; beatriz.reguera@ieo.es (B.R.); jesus.gago@ieo.es (J.G.); francisco.rodriguez@ieo.es (F.R.); 2 Ifremer, DYNECO PELAGOS, 29280 Plouzané, France; mickael.le.gac@ifremer.fr

**Keywords:** *Dinophysis*, nitrate, ammonium, urea, uptake rates, N^15^ incubations, antibiotic treatment

## Abstract

Dinoflagellate species of *Dinophysis* are obligate mixotrophs that require light, nutrients, and prey for sustained growth. Information about their nitrogenous nutrient preferences and their uptake kinetics are scarce. This study aimed to determine the preferred nitrogen sources in cultures of *D. acuminata* and *D. acuta* strains from the Galician Rías Baixas (NW Spain) and to compare their uptake kinetics. Well-fed versus starved cultures of *D. acuminata* and *D. acuta* were supplied with N^15^ labeled inorganic (nitrate, ammonium) and organic (urea) nutrients. Both species showed a preference for ammonium and urea whereas uptake of nitrate was negligible. Uptake rates by well-fed cells of *D. acuminata* and *D. acuta* were 200% and 50% higher, respectively, than by starved cells. Uptake of urea by *D. acuminata* was significantly higher than that of ammonium in both nutritional conditions. In contrast, similar uptake rates of both compounds were observed in *D. acuta*. The apparent inability of *Dinophysis* to take up nitrate suggests the existence of incomplete nitrate-reducing and assimilatory pathways, in line with the paucity of nitrate transporter homologs in the *D. acuminata* reference transcriptome. Results derived from this study will contribute to understand Harmful Algal Blooms succession and differences in the spatio-temporal distribution of the two *Dinophysis* species when they co-occur in stratified scenarios.

## 1. Introduction

Margalef [1] proposed a model, the well-known “Margalef’s Mandala”, with turbulence and concentration of inorganic nutrients as the main axes ordinating phytoplankton species into functional groups. Within the Mandala, a division was observed between phytoplankton species that thrived under high turbulence and nutrient concentrations (e.g., diatoms) and those that would succeed under low turbulence and low nutrient environments (e.g., dinoflagellates). Recently, Glibert [2] proposed an enhanced version of this Mandala, depicting the different phytoplankton functional types along 12 axes. The third axis of her version proposed a division between autotrophic and mixotrophic organisms, since it is now known that most phytoplankton species have a “somehow mixed nutritional behaviour” [3,4].

The term mixotrophy refers to an organism that relies on a combination of phototrophy and phagotrophy [5,6] and integrates a rather heterogenous group of organisms with diverse strategies [7]. Phototrophy in mixotrophs can include the use of light to generate chemical energy (ATP) and photoreductants (NADPH), and it may or may not involve uptake of dissolved N and P because sufficient amount of these elements may be provided by the ingestion of prey [8]. Mixotrophy, a widespread feeding behaviour within *K-strategist* phytoplankters (species with low growth rates that dominate under oligotrophic conditions; [1]), may provide a competitive advantage to endure harsh nutrient-limiting environmental conditions [5,9]. Most phototrophic microalgae, including facultative mixotrophs, can be maintained in laboratory cultures using enriched media with inorganic forms of N, i.e., nitrate [10]. Among the mixotrophs, there are particular cases in which the main N contribution may be obtained from the prey, in this way saving energy to the predator. For example, ~70% of P and N uptake of the haptophyte *Prymnesium parvum* has been reported to originate from its prey under deficiency of these nutrients [11].

*Dinophysis* species harbour “stolen” chloroplasts of cryptophyte origin [12] but such “kleptoplasts” are acquired through a vector organism, the phototrophic ciliate *Mesodinium rubrum* [13]. Phagocytosis of the ciliate prey by *Dinophysis acuminata* was found to contribute to around 50% of its daily carbon intake [14]. The question remained whether nitrogen supply in *Dinophysis* cells comes from dissolved nutrients or is obtained from their prey.

Several field studies indicated a preference for regenerated forms of nitrogen in *Dinophysis acuminata* (ammonium and organic molecules; [15,16,17]). These trends have been confirmed in laboratory incubations of *D. acuminata* [18,19] which yielded no uptake, or fairly low rates, of inorganic forms of N and P, but rapid assimilation of urea. All previous results suggested that *Dinophysis* is either a III B1 type of mixotroph (*sensu* [8]), i.e., “*protists that retain chloroplasts and sometimes other organelles from one algal species or very closely related algal species*”, or a pSNMC (plastidic Specialist Non-Constitutive Mixotroph) [7].

Several species of *Dinophysis* are able to produce one or two groups of lipophilic toxins: (i) okadaic acid (OA) and dinophysistoxins (DTXs), also known as diarrhetic shellfish toxins and (ii) pectenotoxins (PTX) [20,21]. Diarrhetic shellfish toxins cause a gastrointestinal syndrome, Diarrhetic Shellfish Poisoning (DSP), a public health problem for shellfish consumers exposed to *Dinophysis* blooms [22,23]. Shellfish harvesting closures are enforced when toxin levels exceed the regulatory limits (160 μg toxin kg^−1^ flesh), causing considerable economic losses to the shellfish industry [24].

Monitoring of the main toxin-producing species of *Dinophysis*, *D. acuminata* and *D. acuta,* in the Galician Rías Baixas during the last three decades has revealed considerable interannual variability of *Dinophysis* blooms and DSP outbreaks. These two species bloom in different seasons: *D. acuminata* normally occurs from spring until late summer [25,26], whereas *D. acuta* outbreaks are usually the result of advection of allochtonous populations in autumn [27,28]. Exceptionally, the latter may bloom, during very hot summers under a combination of persistent thermal stratification and moderate upwelling [29,30]. Different scenarios, promoting the bloom of one species or the other, have distinct nutrient regimes with contrasting contributions of new (nitrate from nutrient-rich upwelled waters) and regenerated production (ammonium and organic N sources) [31,32,33].

Reasons leading to the interannual and seasonal variability of the two species of *Dinophysis* in the Rías Baixas are not fully understood. Recently, two comparative studies of their responses to variable light and turbulence conditions have been performed [34,35], but their preferences for different nitrogen sources have not been examined so far. This information is of primary importance for the design of predictive models, including nitrogenous nutrient source-preferences, focused on the development of *D. acuminata* and *D. acuta* blooms in the Rías [36,37].

The objectives of the present study were i) to confirm the capacity of *D. acuta* and *D. acuminata* strains to take up dissolved nitrogenous (N) compounds; ii) to identify the preferential N source(s) for each species and iii) to determine interspecific differences in uptake rates of *D. acuminata* and *D. acuta* under well-fed and starved laboratory conditions.

## 2. Materials and Methods 

### 2.1. Culture Conditions

*Dinophysis* cultures were established from water samples collected in the Galician Rías Baixas (NW Spain): *D. acuminata* (VGO1349) was isolated in July 2016 from Ría de Vigo and *D. acuta* (VGO1065) in October 2010 from Ría de Pontevedra. The ciliate *Mesodinium rubrum* (AND-A0711) was isolated in 2007 from Huelva (SW Spain) and the cryptophyte *Plagioselmis prolonga* (CR10EHU), in 2003 from the Bay of Biscay (northern Spain). All cultures were grown in autoclaved seawater enriched with diluted (1:20) L1 (-Si) medium [38] to avoid outgrowth of cryptophytes, at pH 8.0 ± 0.02 and salinity 32. They were kept in a temperature controlled room at 15 ± 1 °C, a 12:12 L:D cycle and an irradiance of ~200 μmol photons m^−2^ s^−1^ PAR provided by Osram Cool White 58W/640 fluorescent tubes. All cultures were non-axenic.

Prior to the experiments, *D. acuminata* and *D. acuta* were grown with diluted (1:20) L1 medium in glass flasks of increasing volumes (from 250 mL to 5 L and fed cryptophyte-free *Mesodinium rubrum* weekly at a 1:10 *Dinophysis:Mesodinium* ratio during 2 months to build up enough biomass for the experiments. After this period, *Dinophysis* cells were washed through a 20-μm mesh, re-suspended in fresh medium and kept separately in 5 L flasks. Autoclaved aged seawater with salinity adjusted to 32 was used for all purposes throughout the pre- and experimental periods, and glassware previously washed with diluted (1:5) HCl, rinsed with distilled water and dried to eliminate organic matter. Whatman GF/F (25 mm Ø, 0.7 μm pore size) glass microfiber filters (Sigma-Aldrich, Munich, Germany) were precombusted at ~450 °C in a muffle oven for 2 h.

### 2.2. Antibiotic Treatments

Initial exposure of *Dinophysis* cultures to a full strength (1:1) filter-sterilized antibiotic cocktail containing ~5000 penicillin units, 5 mg streptomycin and 10 mg neomycin ml^−1^ (Sigma-Aldrich, Germany), equivalent to 186 µL of antibiotic solution per container, brought down the photosynthetic performance (*F_v_/F_m_* = maximum photochemical quantum yield of photosystem II) close to zero. In consequence, a diluted (1:2) concentration that did not decrease *Fv/Fm* values relative to the controls was used.

Fluorescence measurements (*ϕY*(*II*), photochemical quantum yield of the photosystem II) were carried out after 1 h incubation with a PAM (Pulse-Amplitude Modulation) fluorometer to monitor the photosynthetic state of the cells prior to the experiments. Simultaneously, a preliminary test with the same experimental conditions was set up to quantify the bacterial load reduction after the addition of antibiotics. Flow cytometry analyses were carried out with a BD FACS Aria III cell sorter, using triplicate subsamples (15 mL) from cultures of the two species, with and without antibiotics. Nucleic acids were stained using SYBR-green (Molecular Probes, Eugene, OR, USA).

### 2.3. Background Nutrient Concentrations

*Dinophysis* and *Mesodinium* cultures were scaled-up using diluted (1:20) L1 enrichment medium [38], with filtered (0.22 µm) and autoclaved 1-year-aged seawater adjusted to a salinity of 32.

Nutrient concentrations similar to natural seawater conditions in the Rías Baixas during an upwelling event [33,39] were complemented with trace additions of radio-labeled nitrogen isotopes. Ambient concentrations (µmol·L^−1^) of NO_3_^−^, NO_2_, NH_4_^+^, and CO(NH_2_)_2_ in the seawater used for culture media were analyzed in six 50 mL samples (Table 1). 

NO_3_^−^, NO_2_, and NH_4_^+^ concentrations were determined by segmented flow analyses with Futura-Alliance autoanalyzers following [40] and CO(NH_2_)_2_, according to [41]. Analyses were carried out using an UV-2401 PC spectrophotometer Shimadzu (UV-VIS). Urea concentration was estimated by interpolation in the calibration line, the maximum of absorbance being at 524.80 nm. 

### 2.4. N^15^-Radiolabeled Stock Solutions 

Stock solutions with each nitrogen source were prepared with 100 mL of autoclaved seawater, salinity adjusted to 32, with the background nutrient concentrations described in Table 1. Additions of radiolabeled nitrogen sources were based on the difference between the concentrations described on Table 1 and those observed during upwelling events (~10 μmol·L^−1^ for nitrate, ~3.5 μmol·L^−1^ of ammonium and ~0.5 μmol·L^−1^ urea). Thus, tracer additions were 12.89 mg of nitrate (Sodium nitrate-^15^N, 98 atom % ^15^N, Sigma-Aldrich, Germany), 3.70 mg of urea (Urea-^15^N_2_, 98 atom % ^15^N, Sigma-Aldrich, Germany) and 1.08 mg of ammonium (Ammonium-^15^N_2_ sulfate, 98 atom % ^15^N, Sigma-Aldrich, Germany). These solutions were stored in polystyrene bottles of 500 mL, kept in darkness and used for Experiments 1 and 2.

### 2.5. Nitrogen Uptake by Dinophysis acuminata and D. acuta in Well-Fed (Experiment 1) and Starved (Experiment 2) Conditions

#### 2.5.1. Experiment 1

**Figure 1:**
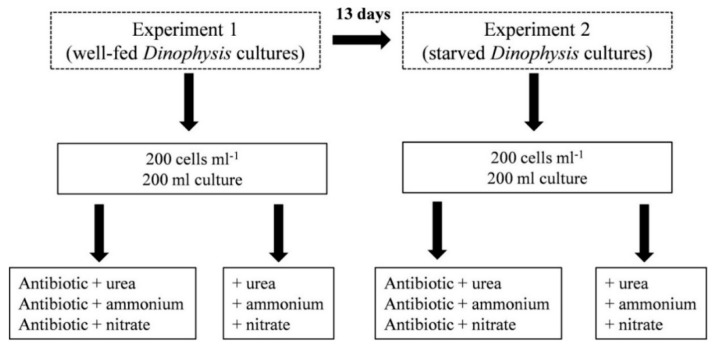
Diagram of the experimental design.

Well-fed washed cells of *Dinophysis* were inoculated, in triplicate, to reach an initial density of 200 cells mL^−1^ (200 mL total volume) in 500 mL polystyrene bottles (Corning, NY, USA) (Figure 1). Cultures were observed under the microscope to check *Mesodinium* cells were not present. After the incubations with antibiotics and PAM measurements, 6 mL of stock solution of the corresponding radiolabeled N compound was added to each treatment. After 3 h incubation and just before the filtration step, subsamples of 1.5 mL for cell counting under an inverted microscope (Nikon Eclipse, TE2000-S, Tokyo, Japan) were collected and fixed with Lugol’s solution. Then, cultures were filtered, the filters dried overnight in an oven at 40 °C and the dried filters kept in Eppendorf tubes.

#### 2.5.2. Experiment 2

*Dinophysis* cells used were inoculated from the stock cultures. The initial conditions and procedures were the same as in Experiment 1. Time elapsed between Experiments 1 and 2 was 13 days (Figure 1). 

### 2.6. Nitrogen Isotope Analysis and Uptake Estimates 

Determination of δ^15^N_AIR_ was carried out through sample combustion at ~1020 °C in an elementary analyser EA 1112-HT (Thermo, Massachusetts, USA). The generated combustion gases were purified and transferred to a Delta VAdvantage (Thermo, Massachusetts, EEUU) isotope ratio mass spectrometer (IRMS) through a Conflo III gas distribution system. International standards used were IAEA N2, IAEA 305-Be IAEA 311. JGOFS [42] protocols based on Dugdale and Goering [43] were used to measure ^15^N isotope tracer levels and estimate N uptake (pmol N cell^−1^ h^−1^) according to:

N uptake (nmol·L^−1^·t^−1^) = (^15^N_xs_ × PN_t_)/(^15^N_enr_ × t) (1)

where t is the incubation time (hours), ^15^N_xs_ is the exceeding ^15^N (measured ^15^N minus ^15^N ambient level, 0.366 atom %) in the post-incubation particle sample, PN_t_ the particulate nitrogen content of the sample after incubation in units of nmol·L^−1^, and ^15^N_enr_ the ^15^N enrichment in the dissolved fraction, estimated according to:

^15^N_enr_ = [(100 × ^15^N)/(^15^N + ^14^N)] − ^15^N_n_ (2)

where ^15^N is the concentration of labeled N, nmol·L^−1^, ^14^N the concentration (same units) of unlabeled N and ^15^N in the ambient level of ^15^N. Cell counts were used in the transformation from nmol·L^−1^·t^−1^ to pmol N·cell^−1^·h^−1^.

### 2.7. Reference Transcriptome of D. acuminata and Other Dinoflagellates

Dinoflagellate reference transcriptomes from the Marine Microeukaryote Transcriptome Sequencing Project (MMETSP; [44]) were downloaded from Cyverse (http://www.cyverse.org). The reference corresponding to *A. minutum* was replaced by the one obtained by Le Gac et al. [45]. The 33 heterotrophic and photosynthetic dinoflagellates, including the only reference transcriptome available for *Dinophysis* (*D. acuminata*) were considered. For each reference transcriptome, a blastx homology analysis (*e*-value < 0.001) was performed against a custom database obtained using protein sequences with “nitrate reductase”, “nitrite reductase”, “nitrate transporter”, “ammonium transporter”, and “urease” in their name from the Uniprot/Swiss-Prot database (https://www.uniprot.org/). For each type of proteins, Fisher Exact were performed to test if the number of homologs (taking into account the length of the transcriptome) in the *D. acuminata* reference transcriptome is similar to what is found overall in the other dinoflagellates.

## 3. Results

### 3.1. Effect of Antibiotics in Dinophysis Cultures

*Dinophysis acuminata* and *D. acuta* cells presented an intense red-pigmentation, normal morphology and swimming behaviour after the addition of a full (1:1) antibiotic solution. However, photosynthetic rates plummeted following *Fv/Fm* measurements (data not shown) and thus, a diluted (1:2) cocktail solution was used instead. *Dinophysis* cultures treated with the diluted solution of antibiotics exhibited a bacterial load reduction of 17.74 ± 1.86% (*n* = 3). 

**Figure 2:**
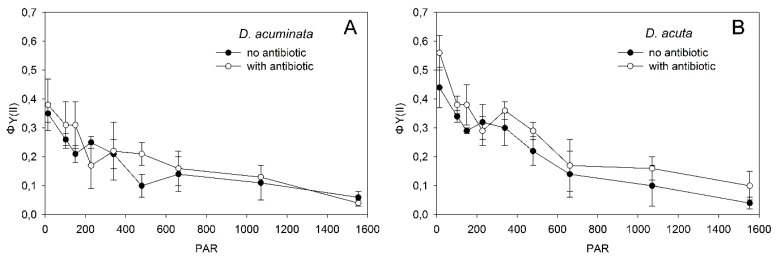
PAM rapid light curves (RLCs) of ΦPSII in **A**) *Dinophysis acuminata* and **B**) *D. acuta* triplicate cultures with (treatments) and without (controls) antibiotics with 8 different PAR conditions. Error bars represent standard deviations (*n* = 3).

Rapid light curves (RLCs) carried out with PAM-fluorometry showed that both cultures, with (treatments) and without (controls) antibiotics, were in good conditions. Changes in *ϕY(II)* values through the eight PAR steps are shown in Figure 2.

### 3.2. Uptake of Different N Sources by D. acuminata and D. acuta

The nutritional status of the cells (well-fed/starved) significantly (*p* < 0.01) influenced the uptake of N sources in both species of *Dinophysis*. Uptake by well-fed cells of *Dinophysis* was always two- to threefold higher than by starved cells. The addition of antibiotics did not significantly affect (*p* > 0.3) the N uptake in any of the species (Table 2).

**Figure 3:**
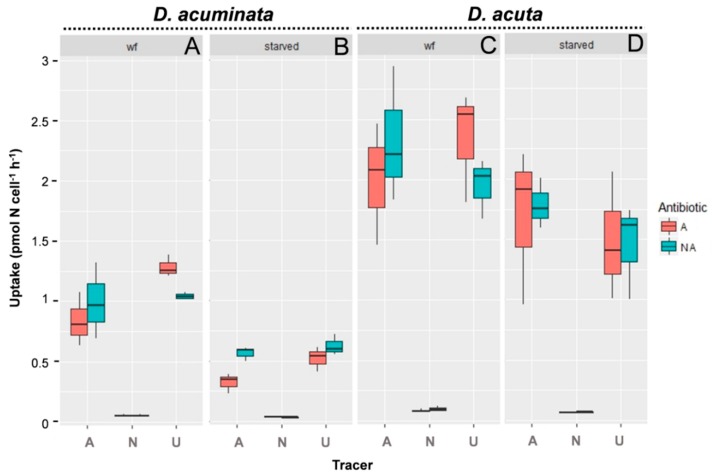
Uptake rates of different radio-labeled nitrogen sources (A: ammonium, N: nitrate and U: urea) by (**A**) well-fed and (**B**) starved cells of *D. acuminata* and by (**C**) well-fed and (**D**) starved cells of *D. acuta*., with antibiotics (A) or without antibiotics (N.A.) added.

In cultures of *D. acuminata*, ammonium and urea uptakes were up to 15–20-fold higher than those of nitrate (*p* < 0.01, Tukey HSD, Table 2), which were almost negligible. Urea was preferred to ammonium and their uptake rates were heavily affected by nutritional status (*p* < 0.01, Tukey HSD, Table 2) (Table S1, Supplementary Materials; Figure 3). Significant differences were observed between well-fed compared with starved cells (*p* < 0.001; Table 2). Uptake of ammonium and urea by well-fed was significantly higher than by starved cells (*p* < 0.001) for urea and ammonium.

In the case of *D. acuta*, uptake rates of ammonium and urea were also significantly higher than the estimated uptake of nitrate (*p* < 0.01, Table 2). However, there were no significant differences between uptake of ammonium and urea (*p* = 0.64, Table 2).

### 3.3. Reference Transcriptome of D. acuminata

Transcripts of transporter and reducer homologs involved in the absorption and assimilation of nitrogen compounds were retrieved from the reference transcriptomes of 33 dinoflagellates (Table S2, Supplementary Materials). Among them, the genus *Dinophysis* is represented by a single species, *D. acuminata*. Three heterotrophic species were also included (*Crypthecodinium cohnii, Noctiluca scintillans*, and *Oxyhrris marina*).

Our results showed that relative to the studied dataset, *D. acuminata* was strongly depleted in nitrate transporter homologs (Table 3; *p* < *0*.01, odd ratio 0.3), accounting for the lowest value, only close to *N. scintillans*. *Dinophysis acuminata* also showed a slight depletion of nitrate reductase (*p* = 0.01, odd ratio 0.75) and ammonium transporter homologs (*p* = 0.03, odd ratio 0.57).

## 4. Discussion

### 4.1. Preferences and Uptake of Nitrogenous Compounds by Dinophysis Species

Earlier studies [15] reported a preference for regenerated forms of N (ammonium, urea) during incubation of field samples of a *D. acuminata* bloom with radiolabeled compounds in the southern Benguela upwelling system. Later, other authors [17] found a positive growth response after addition of 15N labeled ammonium to field samples from Meeting-house Creek and Northport Bay, New York (USA) dominated by *D. acuminata*. A preference for ammonium was also found in *D. acuminata* cultures of a strain isolated from New York bay by the same authors when different 15N labeled nitrogenous sources were provided [19].

There were differences between the experimental setup in Hattenrath-Lehmann and Gobler [19] and the present study. Culture volumes here were four times larger (200 vs. 50 mL), the inoculum twice as dense (200 vs. 100 *Dinophysis* cells·mL^−1^), the incubation time, according to JGOFS [42] protocols, three times higher (3 vs. 1 h), and ^15^N tracer additions the same to well fed than to starved cultures of *D. acuminata* with a background concentration of nutrients similar to those in the Galician Rías during upwelling. In contrast, tracer additions were 180% and 4% of the ambient pools for ammonium, 70% and 140% for nitrate, and 50% and 60% for urea for well fed and starved cultures, respectively, in Reference [19]. These authors referred to Collos and Harrison [46] to suggest a risk of cell intoxication in starved cultures in the presence of high concentrations of ammonium. In this regard, ammonium levels used in the present study (3.5 μmol·L^−1^) were far from the average toxic levels for Dinophyceae (NH4 + NH3 = 1139 ± 2494 μM) reported in Collos and Harrison [46] and also from the values considered to be toxic for *Tripos furca* (20 μM) [47]. Parallel experiments with antibiotics to avoid interference due to bacterial metabolism failed to provide viable axenic cultures of *Dinophysis*. Hattenrath-Lehmann and Gobler [17] assumed a minimal contribution of bacteria during *D. acuminata* incubations, arguing that most of them would be eliminated in the filtration step. These authors observed *Dinophysis* cell mortality after the addition of antibiotics and discarded them in their experiments. In our case, cultures treated with antibiotic showed signs of photoinhibition if treated with full (1:1) or a minor reduction of the bacterial load if treated with diluted (1:2) antibiotic solution.

Despite large methodological differences between the two studies, results from incubations of strains of two species of *Dinophysis* from the Galician Rías with different ^15^N-labeled nitrogen sources qualitatively agreed with those reported by Hattenrath-Lehmann et al. [17] with a strain of *D. acuminata* from New York. *Dinophysis* species from both experiments showed a clear preference for the most reduced (ammonium) inorganic and for the organic (urea) source of N; their nitrate uptake was negligible. Concerning comparison between the two Galician species, *D. acuminata*, whether starved or well-fed, preferred urea to ammonium, whereas *D. acuta* showed similar uptake rates for both nutrients. In quantitative terms, uptake rates for ammonium and urea by *D. acuta*, a species with a cell volume 3.2 higher than *D. acuminata* [48], were proportional to their body size differences.

### 4.2. Uptake Rates of Well-Fed versus Starved Cells of Dinophysis

Nutritional status (well-fed vs. starved) of the cells appeared to be the most relevant (statistically significant) factor to the uptake rate of nitrogenous compounds in *D. acuminata* and *D. acuta* cultures.

There is a considerable body of literature about microalgal nutrient uptake. Nutrient deficiency and starvation have been related to enhanced maximum uptake rate [49], at least on short time scales [50]. These scales are in the range of those used (< 4 h) in the ^15^N incubation experiments. However, the opposite seems to be true in *Dinophysis* cultures. Earlier experiments by Hattenrath-Lehmann and Gobler [19] found a fivefold higher uptake rate of ammonium in well-fed than in starved cells of *D. acuminata* and nearly no difference in the case of urea. The same trend was observed in *Dinophysis acuminata* from the Galician Rías, but rates for ammonium and urea were 2-fold and 6-fold higher, respectively, than those reported for the North American strains.

The observations of declined N uptake rates with starved cells of *Dinophysis*, during short term (2–4 h) incubations with ^15^N, may be explained by their nutritional behavior. Mixotrophic species of *Dinophysis* are obligate mixotrophs requiring light and prey for sustained growth [51]. In a recent classification, they were labeled as “plastidic specialists non-constitutive mixotroph” (pSNCM) requiring the plastids of a specific prey [7], the ciliate *Mesodinium*, itself a kleptoplastidic phototroph requiring cryptophyte flagellates belonging to the TPG (*Teleaulax*/*Plagioselmis*/*Geminigera*) clade [52]. Minnhagen et al. [53] concluded that starved cells of *Dinophysis* have a reduced photosynthetic capacity because their kleptoplastids pool gets diluted as cell division continues for 3–5 generations without acquisition of new prey. Fritz et al. [54], using nitrate reductase immunolocalization with the dinoflagellate *Lingulodinium polyedrum*, showed that nitrate reduction was carried out in the chloroplasts. It seems plausible to expect that a smaller number of functionally impaired plastids in starved cells of *Dinophysis* would bring down their N assimilation capacity and biosynthetic activity. This view is supported by observations of a gradual decline of the photosynthetic performance in old kleptoplastids relative to that of the newly acquired in well-fed cells [55]. Recently, Rusterholz et al. [56] rejected the “dilution” theory and suggested a certain capacity of *Dinophysis* for kleptoplastid division, arguing in favour of a greater control than previously thought. In any case, starved condition led to a decline in kleptoplastid numbers per cell.

### 4.3. Why D. acuminata and D. acuta Do Not Take Up Nitrate?

The reduced number of nitrate transporter homologs detected in the single reference transcriptome of *D. acuminata*, much lower than in the rest of dinoflagellates in the dataset (with the exception of the heterotroph *Noctiluca scintillans*), points to poor nitrate transport into the cell, in agreement with culture and field observations, and potentially to the lack of a complete nitrate-reducing and assimilatory pathway.

In photosynthetic protists, nitrate assimilation is an active process performed first by transport into the cells and then by subsequent reduction steps. The enzyme Nitrate Reductase (NR) catalyzes the reduction step to nitrite, and the Nitrite Reductase (NiR) its reduction to ammonium ([57] and references therein). Urea is turned into NH_3_^+^/NH_4_^+^, released and assimilated inside the cell [58]. Ammonium (NH_4_^+^) is the easiest N form for dinoflagellates (and other primary producers) to acquire and transport into the cell since these processes require less energy [58,59], but concentrations above 25–50 µm may be toxic for some species [60,61,62].

The absence of most plastid-related genes in *Dinophysis acuminata* may be the reason this species cannot keep kleptoplasts permanently [63]. As illustrated in the present study, the depletion of nitrate metabolism related transcripts may explain its inability to assimilate nitrate. Taken together, while most dinoflagellates with permanent chloroplasts are able to grow if supplied strictly with inorganic nutrients, e.g., nitrate [10], mixotrophic organisms like *Dinophysis* can take up different proportions of N either by transporters or through heterotrophic feeding [64], thus compensating for the apparent inability to incorporate nitrate. It is already known that prey capture in *Dinophysis* provides an outstanding source of energy, and according to [14], that they need to cover about half of their carbon demand from prey ingestion for optimal growth. The proportions of N incorporated by each mechanism (photosynthesis versus phagotrophy) are unknown but it can be hypothesized that prey capture in *Dinophysis* is more relevant to this purpose than N transporters. This view is supported by the depletion of nitrate- and nitrite-reductase, but even more, by the low number of nitrate and ammonium transporter homologs (Table 3).

### 4.4. Ecological Implications

*Dinophysis* strains used in this study are from the Galician Rías Baixas (Muros, Arousa, Pontevedra, and Vigo), northernmost limit of the Canary Current Upwelling System and site of intensive raft-mussel cultivation (see, e.g., [65]). Upwelling events in spring and summer inject cold nitrate-rich waters into the Rías controlling nutrients and phytoplankton dynamics [66,67]; suspended mussels excrete plenty of ammonium and urea into the water column which is exploited by the phytoplankton community [68]. Large amounts of particulate matter are flushed out of the Rías in the upwelling season, and from sediments in the adjacent shelf where intense remineralization processes take place, in particular in summer; these regenerated nutrients may be reintroduced to the Rías with the shoreward upwelling currents. Nearly 70% of the mussel production (2.5 × 10^6^ t per year) takes place in Ría de Arousa, but morphology and bathymetry of this large ría favours more intense upwelling, shorter water residence times, maximal primary production, and minimal duration of harvesting closures for DSP toxins in the region [69]. Results from incubations with radiolabelled ^15^N during blooms of different toxin-producing microalgae in the Benguela and the Canary Current upwelling allowed Seeyave et al. [15,16] to identify different nutrient acquisition strategies. Some species, such as *Pseudo-nitzschia* spp. (pennate diatoms) and the dinoflagellate *Alexandrium catenella*, which take up nitrate at a high rate, were classified as “velocity strategists”, well adapted to exploit the new production from recently upwelled waters. In contrast, *D. acuminata*, with the highest affinity for ammonium, was considered an “affinity strategist”, with adaptations to thrive in nitrogen-depleted waters. These observations agree with previous field observations and modeling simulations showing that the annual dinoflagellates maxima, including *Dinophysis* spp., occurred when there was a predominance of regenerated production [31,70]. This situation is common during downwelling at the end of the spring-summer upwelling season (the autumn transition) or within the short time scale (5–15 d) of upwelling-downwelling cycles in spring-summer [71].

Other authors (i.e., Reference [17]) added inorganic and organic radiolabelled N sources to incubations of field populations at the time of *D. acuminata* blooms in New York coastal waters. In microcosm experiments, *Dinophysis* populations increased after the addition of ammonium, urea, concentrated DON, and to a far lesser extent, nitrate. These results led them to conclude that N-nutrients loading promoted growth and toxicity of *D. acuminata*.

Field observation in the rías and the moderate uptake rates of N-compounds by *Dinophysis* species found in the present study support the views of Seeyave et al. [15]. Injection of nutrients following upwelling pulses triggers blooms of large centric diatoms followed by pennate diatoms [72,73]. When blooms of *Pseudo-nitzschia* co-occur with low biomass blooms of *D. acuminata*, they are vertically segregated: *Pseudo-nitzschia* cell maxima are in the pycnocline/nitrocline (~10 m) and occasionally form thin layers, whereas the highest numbers of *D. acuminata* appear near the surface (2–5 m) (see, e.g., [74,75]). It seems highly improbable that *Dinophysis* species can outcompete diatoms in a N-nutrients uptake race. Their strategy relies on a combination of ciliate (*Mesodinium* spp) prey availability and hydrodynamic conditions favouring stratification. In fact, increased water residence times in the rías, related to a decline of upwelling-promoting northerly winds and higher water column stability, have been proposed as the key factors behind increased shellfish harvesting closures due to toxin contamination in the early 2000s [67]. A thermally stratified water column during July-August is also related with subsequent summer outbreaks of *D. acuta* in the Rías [30]. Therefore, physical factors (hydrodynamics) control the development, maintenance and decline of *Dinophysis* blooms in the Galician Rías. Vertical segregation of *D. acuminata* and *D. acuta* in the Galician Rías (the former close to the surface and the latter in the pycnocline) [75], and similar observations in *D. acuminata* and *D. norvegica* populations in the Baltic Sea [76], cannot be solely explained in terms of nutrient distributions alone. Attention must be paid to the match/mismatch of the different suites of environmental conditions (irradiance, turbulence, nutritional sources), optimal for *Dinophysis* and for its prey, the ciliate *Mesodinium*.

## 5. Conclusions

*Dinophysis acuminata* and *D. acuta* exhibited a preference for regenerated inorganic (ammonium) and organic (urea) sources of N during incubations of cultures of the two species in well-fed and starved conditions. Uptake rate of these N forms was reduced during starvation, in contrast with the typical uptake enhancement observed after refeeding food-limited microalgae. This is presumably due to weak kleptoplastid performance of prey–starved cells of mixotrophic species of *Dinophysis*. Screening of the different genes involved in N assimilation revealed a strong depletion of nitrate transporters in the *D. acuminata* reference transcriptome compared with other 32 dinoflagellates (29 photosynthetic and 3 heterotrophic ones). This is in agreement with our experimental data and with previous studies about the very low uptake of nitrate or even the lack of it and the high affinity for ammonium and urea. This is expected to be the case also for *D. acuta* and other members of this genus. These findings may help to explain the vertical segregation observed in field populations of *Dinophysis* in relation to the diatom populations during upwelling events. The reasons underlying these findings would merit new studies both in field and culture conditions to improve our understanding of the autoecology of these dinoflagellates and the possible evolution of their populations in the future.

## Figures and Tables

**Table 1:** Background nutrient concentrations (µmol·L^−1^) in the seawater used in Experiments 1 and 2.

Label	NO_3_^−^	NO_2_	NH_4_^+^	PO_4_^3−^	SiO_2_	CO(NH_2_)_2_
1	2.58	0.68	0.96	0.22	5.21	0.14
2	2.08	0.56	0.81	0.18	4.25	0.12
3	2.23	0.60	0.85	0.18	4.43	0.08
4	3.28	0.74	1.04	0.25	6.56	0.08
5	2.30	0.51	0.77	0.19	4.57	0.08
6	2.69	0.62	0.89	0.21	5.41	0.12

**Table 2:** Statistical significance of uptake rates of N sources by *D. acuminata* and *D. acuta* in well-fed and starved conditions, with and without antibiotics. 2w-ANOVA: two-way ANOVA. Asterisks (*) indicate high statistical significance.

Parameters	Statistical Test	*D. acuminata*		*D. acuta*	
		F-Value	*p*-Value	F-Value	*p*-Value
Tracer	2w-ANOVA	147.21	3 × 10^−14^*	89.06	8 × 10^−12^*
Nutritional state	2w-ANOVA	76.56	24 × 10^−9^*	8.63	7 × 10^−3^*
Antibiotic	2w-ANOVA	0.99	0.33	0.00	0.99
Tracer:antibiotic	2w-ANOVA	3.73	0.04	0.93	0.41
Tracer:nutritional state	2w-ANOVA	17.31	2 × 10^−5^*	2.20	0.13
Antibiotic:nutritional state	2w-ANOVA	2.86	10^−1^	0.02	0.89
Nitrate-ammonium	TukeyHSD		10^−7^*		10^−7^*
Urea-ammonium	TukeyHSD		3 × 10^−3^*		0.64
Urea-nitrate	TukeyHSD		10^−7^*		10^−7^*
No antibiotic:antibiotic	TukeyHSD		0.33		0.99
Urea: well-fed-starved cells	TukeyHSD		3 × 10^−7^*		0.05
Ammonium: well-fed-starved cells	TukeyHSD		1 × 10^−5^*		0.41
Nitrate: well-fed-starved cells	TukeyHSD		0.99		0.99

**Table 3:** Number of nitrate and nitrite reductase, nitrate and ammonium transporter, and urease homologs in the *D. acuminata* reference transcriptome. See methods for statistical analysis against a dataset of 32 dinoflagellates (*p* < 0.001).

Protein Family	*D. acuminata*	Odd Ratio	*p*-Value
Nitrate reductase	77	0.75	0.01
Nitrite reductase	13	0.65	0.14
Nitrate transporter	8	0.3	4 × 10^−5^
Ammonium transporter	15	0.57	0.03
Urease	42	0.95	0.8

## Supplementary Materials

The following are available online at https://www.mdpi.com/2076-2607/8/2/187/s1.
