# Supplementary material for: Uptake of Inorganic and Organic Nitrogen Sources by Dinophysis acuminata and D. acuta
Source: Microorganisms. 2020 Jan 29;8(2):187. doi: 10.3390/microorganisms8020187 (PMC7074736; doi:10.3390/microorganisms8020187)
Supplement: Supplementary file 1 [file microorganisms-08-00187-s001.pdf]

Species	N source	Antibiotic	FED		STARVED	
			AV	SD	AV	SD
D. acuminata	Nitrate	+A	0.05	0.01	0.03	0.00
		N.A.	0.05	0.01	0.02	0.02
	Ammonium	+A	0.84	0.22	0.32	0.08
		N.A.	0.99	0.32	0.56	0.06
	Urea	+A	1.28	0.09	0.52	0.10
		N.A.	1.04	0.03	0.62	0.08
D. acuta	Nitrate	+A	0.08	0.01	0.06	0.00
		N.A.	0.10	0.02	0.07	0.01
	Ammonium	+A	2.00	0.51	1.69	0.65
		N.A.	2.33	0.56	1.78	0.21
	Urea	+A	2.34	0.47	1.49	0.53
		N.A.	1.95	0.25	1.45	0.39

Table S1. Uptake rates (pmol N cell-1 h-1) of N sources (urea, ammonium and nitrate) by D. acuminata and D. acuta cells in well-fed and starved conditions. +A: with antibiotic, N.A.: no antibiotic, AV: average, SD: standard deviation.

Species	Nitrate reductase	Nitrite reductase	Nitrate transporter	Ammonium transporter	Urease	Transcripts in ref
A_andersonii	80	15	15	10	27	101059
A_carterae	68	25	20	13	30	72029
A_catenella	92	11	22	19	37	113342
A_margalefii	116	26	23	27	56	135117
A_massartii	61	24	22	32	30	74135
A_minutum	52	8	32	10	36	142770
A_monilatum	145	33	24	29	78	161890
A_spinosum	131	28	31	26	81	208533
A_tamarense	185	33	35	42	69	216202
*C_cohnii**	106	11	28	16	25	129408
D_acuminata	**77**	**13**	**8**	**15**	**42**	**119662**
D_baltica	179	45	52	63	57	171517
G_australes	79	19	26	35	46	96540
G_catenatum	95	33	31	10	50	114447
G_spinifera	73	15	18	17	42	77306
H_arctica	63	19	21	14	29	74907
H_rotundata	64	16	16	25	26	72882
H_triquetra	95	24	18	31	25	95540
K_brevis	299	35	106	52	142	372125
K_foliaceum	316	74	87	150	105	310847
K_veneficum	166	25	50	60	80	184831
L_polyedrum	150	31	48	41	99	179048
*N_scintillans**	68	9	9	11	19	61180
*O_marina**	204	35	44	32	89	243555
P_aciculiferum	123	17	23	10	52	131791
P_bahamense	107	29	18	28	58	141678
P_beii	92	24	34	24	49	103458
P_glacialis	90	19	19	28	31	109183
P_minimum	154	34	36	39	68	222953
P_reticulatum	73	16	30	33	38	106046
S_hangoei	245	24	50	29	73	236636
S_trochoidea	217	23	49	71	72	211069
T_jolla	65	12	30	17	29	70846

Table S2. Number of nitrate reductase, nitrite reductase, nitrate transporter, ammonium transporter, and urease homologs, as well as the total number of transcripts in the reference transcriptomes of 33 dinoflagellates. Asterisks (*) indicate heterotrophic species.
